# Estimation of Cardiovascular Risk Predictors from Non-Invasively Measured Diametric Pulse Volume Waveforms via Multiple Measurement Information Fusion

**DOI:** 10.1038/s41598-018-28604-6

**Published:** 2018-07-11

**Authors:** Zahra Ghasemi, Jong Chan Lee, Chang-Sei Kim, Hao-Min Cheng, Shih-Hsien Sung, Chen-Huan Chen, Ramakrishna Mukkamala, Jin-Oh Hahn

**Affiliations:** 1 Department of Mechanical Engineering, University of Maryland, College Park, USA; 2 School of Mechanical Engineering, Chonnam National University, Gwangju, South Korea; 3 Department of Medicine, National Yang-Ming University, Taipei City, Taiwan; 4 Department of Electrical and Computer Engineering, Michigan State University, East Lansing, USA

## Abstract

This paper presents a novel multiple measurement information fusion approach to the estimation of cardiovascular risk predictors from non-invasive pulse volume waveforms measured at the body’s diametric (arm and ankle) locations. Leveraging the fact that diametric pulse volume waveforms originate from the common central pulse waveform, the approach estimates cardiovascular risk predictors in three steps by: (1) deriving lumped-parameter models of the central-diametric arterial lines from diametric pulse volume waveforms, (2) estimating central blood pressure waveform by analyzing the diametric pulse volume waveforms using the derived arterial line models, and (3) estimating cardiovascular risk predictors (including central systolic and pulse pressures, pulse pressure amplification, and pulse transit time) from the arterial line models and central blood pressure waveform in conjunction with the diametric pulse volume waveforms. Experimental results obtained from 164 human subjects with a wide blood pressure range (systolic 144 mmHg and diastolic 103 mmHg) showed that the approach could estimate cardiovascular risk predictors accurately (r ≥ 0.78). Further analysis showed that the approach outperformed a generalized transfer function regardless of the degree of pulse pressure amplification. The approach may be integrated with already available medical devices to enable convenient out-of-clinic cardiovascular risk prediction.

## Introduction

Cardiovascular disease (CVD) is the most prevalent chronic disease in the United States and around the world^1^. It has been well known that CV health and disease may be assessed by means of an array of CV risk predictors, including central systolic and pulse pressures (SP and PP), aortic PP amplification, and aortic pulse wave velocity (PWV)^2–7^. However, some of the state-of-the-art techniques for estimating gold standard CV risk predictors are inconvenient and costly. For example, the measurement of central SP and PP requires aortic catheterization^7–11^ (which is invasive) or carotid artery tonometry^12,13^ (which involves costly probe and trained operators). The measurement of aortic PP amplification and PWV (or equivalently, pulse transit time (PTT)) likewise necessitates inconvenient carotid-femoral tonometry procedure^5,14–18^. It is arguable that these cost and convenience issues have hampered early detection and timely treatment of CVD. Hence, novel technologies to complement the state-of-the-art CV health and risk predictors estimation techniques via convenient out-of-clinic monitoring and tracking of CV risk predictors may significantly improve the prevention, early detection, and treatment of CVD.

Effort has been made to enable more convenient and affordable estimation and tracking of CV risk predictors based on automated cuff devices^19–26^. In this approach, pulse volume pulsation (called the pulse volume recording (PVR) in the literature) waveform of the brachial artery is measured while cuff pressure is maintained at a constant sub-diastolic or supra-systolic level. The PVR waveform is then calibrated to the arm cuff BP measurement and converted to central BP waveform using a mathematical transformation. However, the vast majority of the prior work have two shortcomings: (1) the techniques rely on population-based transformation (called hereafter the generalized transfer function (GTF)) to convert the arm PVR waveform to central BP waveform and thus may not achieve optimal accuracy; and (2) CV risk predictors other than central BP, especially those associated with the central aorta (such as aortic PP amplification and PWV), cannot be readily estimated due to the absence of relevant distal aortic pulse measurement.

In a series of prior work, we have demonstrated that CV risk predictors including central BP^10,27–29^, PTT^30^, and wave reflection characteristics^31^ may be estimated in a subject-specific fashion from arterial pulse waveforms measured at the body’s diametric (i.e., arm and leg) locations. This so-called multiple measurement information fusion approach is built on the novel idea that diametric arterial pulse waveforms, despite their difference in morphology (due to the difference in the arterial line dynamics associated with each of the diametric locations), originate from common central BP, and therefore, central BP may be inferred from judicious analysis of the diametric arterial pulse waveforms. To date, the validity of this multiple measurement information fusion approach has been limited to the use of invasive diametric BP waveforms. Yet, considering already available dual-cuff devices^23,32–34^, this approach has potential to be integrated with such devices to yield convenient, affordable, and subject-individualized technologies for estimation and tracking of CV risk predictors.

This paper presents a novel multiple measurement information fusion approach to the estimation of CV risk predictors from non-invasive pulse volume (PVR) waveforms measured at the body’s diametric locations. Leveraging the fact that diametric PVR waveforms originate from the common central pulse waveform, the approach estimates CV risk predictors in three steps by: (1) deriving lumped-parameter models of the central-diametric arterial lines from diametric PVR waveforms, (2) estimating central BP waveform by analyzing the diametric PVR waveforms using the derived arterial line models, and (3) estimating CV risk predictors (including central SP and PP, PP amplification, and PTT) from the arterial line models and central BP waveform in conjunction with the diametric PVR waveforms. In this study, we report the results of investigating the performance of this novel approach using the experimental data collected from 164 subjects.

## Methods

### Experimental Data

We studied electronically archived data from 164 human subjects that were originally obtained in a previous study under the approval from the institutional review board at the Taipei Veterans General Hospital and written informed consent^35^. The study was performed in accordance with relevant guidelines and regulations. These subjects had central BP waveform measured at the carotid artery using the applanation tonometry, as well as PVR waveform recordings from the upper arm (i.e., brachial artery) and ankle (i.e., posterior-tibial artery) using automated arm and ankle cuff devices. The carotid BP and PVR waveforms were calibrated using the brachial mean (MP) and diastolic (DP) BPs obtained using the oscillometric BP measurement method. We randomly split the 164 subjects into 50 for training and 114 for testing of the proposed multiple measurement information fusion approach to estimation of CV risk predictors from diametric PVR waveforms, which is an approximate proportion of 1:2.

To investigate the efficacy of the multiple measurement information fusion approach to the estimation of CV risk predictors, the following CV risk predictors were computed from both training and testing data and used as reference CV risk predictors. First, reference central SP and PP were computed as the carotid SP and PP. Second, reference aortic PP amplification was computed as the ratio of carotid PP and ankle PP. Third, reference aortic PTT was computed as the time delay between the diastolic troughs of the carotid BP and ankle PVR waveforms^36^.

### Estimation of CV Risk Predictors from Diametric PVR via Multiple Measurement Information Fusion

**Figure 1:**
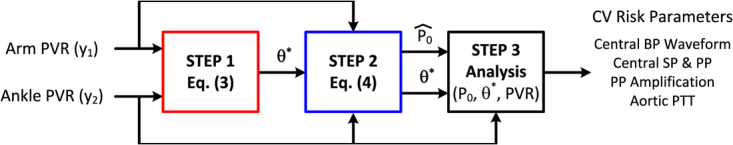
Overview of multiple measurement information fusion approach to estimation of cardiovascular (CV) risk predictors. The approach estimates CV risk predictors in three steps. Step 1: Lumped-parameter models of the central-diametric arterial lines are derived from diametric pulse volume (PVR) waveforms using Eq. (3). Step 2: Central blood pressure (BP) waveform is estimated by analyzing the diametric PVR waveforms using the derived arterial line models using Eq. (4). Step 3: CV risk predictors (including central systolic and pulse pressures, pulse pressure amplification, and pulse transit time) are estimated from the arterial line models and central BP waveform in conjunction with the diametric PVR waveforms.

Figure 1 illustrates the proposed multiple measurement information fusion approach to estimation of CV risk predictors from diametric PVR waveforms. The approach estimates CV risk predictors in three steps. First, lumped-parameter models of the central-diametric arterial lines are derived from diametric PVR waveforms. Second, central BP waveform is estimated by analyzing the diametric PVR waveforms using the derived arterial line models. Third, CV risk predictors (including central BP waveform, central SP and PP, and aortic PP amplification and PTT) are estimated from the arterial line models and central BP waveform in conjunction with the diametric PVR waveforms.

**Figure 2:**
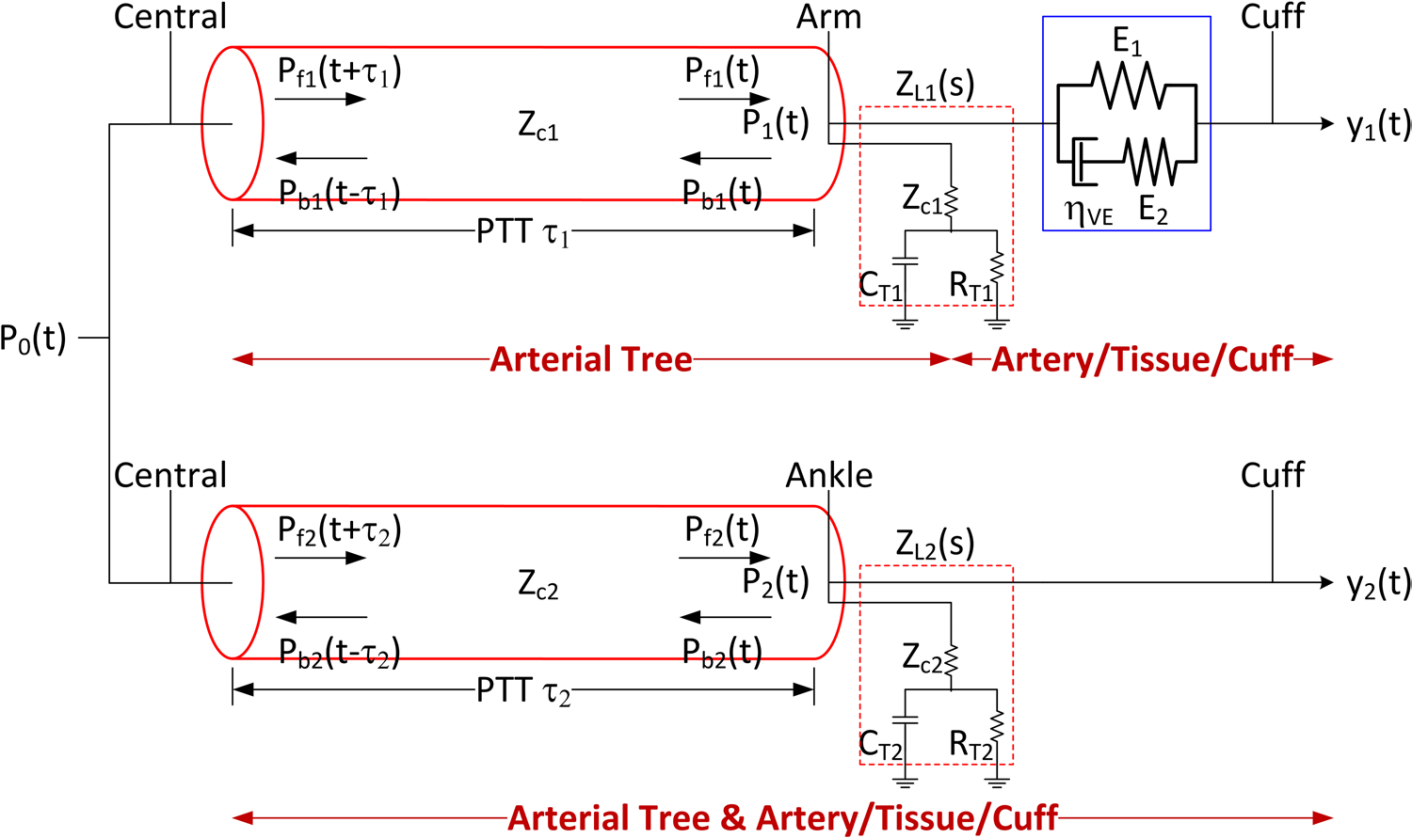
Lumped-parameter (tube-load) models to relate central blood pressure (BP) waveform to diametric pulse volume (PVR) waveform. Each arterial tree model has a constant characteristic impedance Z_Cj_ (j = 1,2) and allows BP wave to travel with a constant pulse wave velocity (PWV). Pulse transit time (PTT) τ_j_ (j = 1,2) is given by dividing the central-diametric (arm or ankle) arterial distance by PWV. The model of the terminal load (Z_Lj_ (j = 1,2)) includes the resistances Z_Cj_ (j = 1,2) and R_Tj_ (j = 1,2) as well as compliance C_Tj_ (j = 1,2) of the distal arteries. Each BP waveform (P_0_, P_1_, and P_2_) are given by the sum of forward (P_fj_(t), j = 1,2) and backward (P_bj_(t), j = 1,2) BP waves at the corresponding location: P_j_(t) = P_fj_(t) + P_bj_(t), j = 1,2, and P_0_(t) = P_fj_(t + τ_j_) + P_bj_(t − τ_j_), j = 1,2.

The lumped-parameter central-diametric arterial line models are derived from diametric PVR waveforms by leveraging the fact that diametric PVR waveforms originate from the common central pulse waveform from the heart. Using the following tube-load models developed and validated in our prior work^37^ to relate central BP waveform (*P*_0_) to the arm (*y*_1_) and ankle (*y*_2_) PVR waveforms (Fig. 2):

1 $$\begin{array}{c}{y}_{1}(s)={G}_{1}(s){P}_{0}(s)=\frac{{E}_{2}+{\eta }_{VE}s}{{E}_{1}{E}_{2}+({E}_{1}+{E}_{2}){\eta }_{VE}s}\frac{s+{\eta }_{11}+{\eta }_{21}}{{e}^{{\tau }_{1}s}(s+{\eta }_{11})+{e}^{-{\tau }_{1}s}{\eta }_{21}}{P}_{0}(s)\\ {y}_{2}(s)={G}_{2}(s){P}_{0}(s)=\frac{s+{\eta }_{12}+{\eta }_{22}}{{e}^{{\tau }_{2}s}(s+{\eta }_{12})+{e}^{-{\tau }_{2}s}{\eta }_{22}}{P}_{0}(s)\end{array}$$

where *s* is the Laplace operator, *τ*_1_ and *τ*_2_ are the central-arm and central-ankle PTTs, respectively, *η*_*ij*_, *j* = 1, 2 are the polynomial parameters associated with the *y*_*j*_(*s*), while *E*_1_, *E*_2_, and *η*_*VE*_ are the parameters characterizing the viscoelastic model associated with the brachial artery-tissue-arm cuff interface, the following correlation equation can be formed by canceling the unknown yet common central BP *P*_0_ from Eq. (1):

2 $${G}_{1}^{-1}(s){y}_{1}(s)={G}_{2}^{-1}(s){y}_{2}(s)$$

which can be solved to derive the unknown subject-specific parameters in the arterial line models *G*_1_(*s*) and *G*_2_(*s*) via, e.g., numerical optimization in the time domain^10,38^:

3 $$\begin{array}{ccc}{\theta }^{\ast } & = & \arg \,\mathop{min}\limits_{\theta }\{\parallel {{\mathscr{L}}}^{-1}[{G}_{1}^{-1}(s,\theta ){y}_{1}(s)-{G}_{2}^{-1}(s,\theta ){y}_{2}(s)]\parallel \\  &  & +\,\parallel \,max({{\mathscr{L}}}^{-1}[{G}_{1}^{-1}(s,\theta ){y}_{1}(s)])-\,max({{\mathscr{L}}}^{-1}[{G}_{2}^{-1}(s,\theta ){y}_{2}(s)])\parallel \\  &  & +\,\parallel (max({{\mathscr{L}}}^{-1}[{G}_{1}^{-1}(s,\theta ){y}_{1}(s)])-\,min({{\mathscr{L}}}^{-1}[{G}_{1}^{-1}(s,\theta ){y}_{1}(s)]))\\  &  & -\,(max({{\mathscr{L}}}^{-1}[{G}_{2}^{-1}(s,\theta ){y}_{2}(s)])-\,min({{\mathscr{L}}}^{-1}[{G}_{2}^{-1}(s,\theta ){y}_{2}(s)]))\parallel \}\end{array}$$

where ${\mathcal L}$ is the Laplace transform operator and $\theta =\{{\tau }_{1},{\tau }_{2},{\eta }_{11},{\eta }_{21},{\eta }_{12},{\eta }_{22},{E}_{1},{E}_{2},{\eta }_{VE}\}$ is the vector of unknown arterial line model parameters. Note that the first, second, and third terms in Eq. (3) represent central BP waveform, SP, and PP errors, respectively. Since Eq. (3) is solved using *y*_1_ and *y*_2_ measured from a specific subject, θ^*^ derived from Eq. (3) is specific to the subject at the time of the PVR measurement. It must be noted that multiple measurement information fusion approach requires *G*_1_(*s*) and *G*_2_(*s*) to be distinct for Eq. (2) to be non-trivial. Considering that arterial line dynamics associated with diametric locations are expected to be highly distinct from each other, it is preferable to employ diametric PVR waveforms in realizing the multiple measurement information fusion approach.

The central BP waveform specific to the subject can be estimated from *y*_1_ and *y*_2_ and the subject-specific arterial line models as follows:

4 $${\hat{P}}_{0}(s)={\sigma }_{1}{\hat{P}}_{01}(s)+{\sigma }_{2}{\hat{P}}_{02}(s)={\sigma }_{1}{G}_{1}^{-1}(s,{\theta }^{\ast }){y}_{1}(s)+{\sigma }_{2}{G}_{2}^{-1}(s,{\theta }^{\ast }){y}_{2}(s)$$

where ${\hat{P}}_{0}(s)$ denotes the estimated central BP waveform, ${\hat{P}}_{01}(s)={G}_{1}^{-1}(s,{\theta }^{\ast }){y}_{1}(s)$ and ${\hat{P}}_{02}(s)={G}_{2}^{-1}(s,{\theta }^{\ast }){y}_{2}(s)$ are the central BP waveforms estimated from *y*_1_ and *y*_2_, respectively, and $0\le {\sigma }_{1},{\sigma }_{2}\le 1$ are the weights (note that ${\sigma }_{1}+{\sigma }_{2}=1$).

The CV risk predictors can then be estimated as follows. Central SP and PP can be estimated as the maximum and amplitude values associated with ${\hat{P}}_{0}$. Aortic PP amplification can be estimated as the ratio of estimated central PP and the amplitude value associated with the ankle PVR waveform. Aortic PTT can be estimated as ${\tau }_{2}^{\ast }$ obtained from Eq. (3).

### Data Analysis

**Figure 3:**
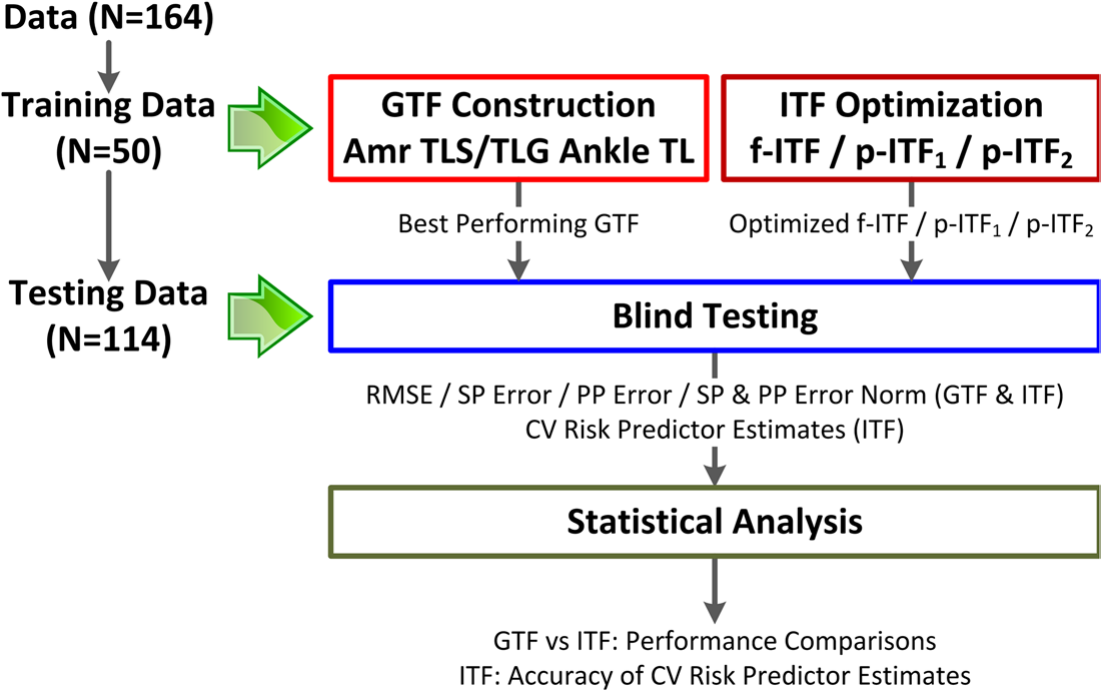
Data analysis procedure.

Using CV risk predictors derived from carotid artery tonometry and ankle PVR waveform measurements as reference, we investigated the performance of the multiple measurement information fusion approach to the estimation of CV risk predictors from non-invasive diametric PVR waveforms in comparison with the state-of-the-art population-based GTF approach (Fig. 3). We used the training data to optimize the multiple measurement information fusion approach outlined in the Estimation of CV Risk Predictors from Diametric PVR via Multiple Measurement Information Fusion section as well as to construct a GTF. Then, we used the testing data to conduct a blind testing and comparatively investigate the efficacy of the multiple measurement information fusion approach and the GTF approach. Note that the GTF constructed in this way enables more rigorous evaluation of the proposed multiple measurement information fusion approach than the GTFs reported in the literature^39^; indeed, the former represents the population considered in this study better than the latter and is thus expected to outperform the latter. Details follow.

We considered a set of GTFs associated with the carotid BP and diametric PVR waveform measurements: an arm GTF based on the tube-load model with a standard linear solid model (taking the form of *G*_1_(*s*) in Eq. (1) with population-averaged parameters, called hereafter the arm TLS GTF), an arm GTF based on the tube-load model with a static gain (taking the form of *G*_1_(*s*) in Eq. (1) with *η*_*VE*_ = 0 and population-averaged parameters, called hereafter the arm TLG GTF), and an ankle GTF based on the tube-load model (taking the form of *G*_2_(*s*) in Eq. (1) with population-averaged parameters, called hereafter the ankle TL GTF). Each GTF was constructed using the training data by first deriving the corresponding arterial line model parameters associated with each of the 50 training subjects from the subject’s carotid BP and PVR waveforms via numerical optimization (similarly to our prior work)^37^, and then implementing the same arterial line model characterized by the median values of the model parameters derived for the 50 training subjects. We compared these GTFs in terms of their accuracy in estimating central BP waveform from the respective PVR waveforms, including the root-mean-squared waveform error (RMSE), absolute SP and PP errors (*e*_*SP*_ and *e*_*PP*_), and SP and PP error norm $(=\sqrt{{e}_{SP}^{2}+{e}_{PP}^{2}})$ across the 50 training subjects. We used the best-performing GTF as the reference technique in investigating the performance of the multiple measurement information fusion approach in the blind testing stage.

We likewise considered a set of realizations of the multiple measurement information fusion approach (called hereafter the individualized transfer functions (ITFs)). First, we optimized the fully individualized transfer function described in the Estimation of CV Risk Predictors from Diametric PVR via Multiple Measurement Information Fusion section (f-ITF, in that all the elements in *θ* in Eq. (3) are individualized) using the training data, in terms of its configurable factors in Eqs (3) and (4) including the parametric search bounds as well as the weights *σ*_1_ and *σ*_2_, so that its accuracy in estimating central BP waveform from diametric PVR waveforms (including the RMSE, absolute SP and PP errors, and SP and PP error norm) was maximized across the 50 training subjects. Specifically, all the parameters in *θ* were restricted to be positive in solving Eq. (3) to preserve their physical implications, while ${\sigma }_{1}={\sigma }_{2}=1/2$ was used in Eq. (4); although not shown, the quality of the estimated central BP waveform ${\hat{P}}_{0}(s)$ was not sensitive to the choice of *σ*_1_ and *σ*_2_. Second, we constructed two partially individualized transfer functions (p-ITFs, in that only a subset of the elements in *θ* in Eq. (3) are individualized) based on the optimized f-ITF, in order to create individualized transfer functions equipped with improved performance and robustness by relaxing the complexity of the optimization problem in Eq. (3), thereby avoiding high-variance model parameters as well as overfitting. One was constructed by fixing the model parameter(s) which exhibited the smallest inter-individual variability across the 50 training subjects (called hereafter the p-ITF_1_), while the other was constructed by fixing the polynomial parameters *η*_11_ and *η*_21_ in *G*_1_(*s*), which are known to often have little impact on the transfer function and may thus be fixed at appropriate nominal values^10,27^. We derived these p-ITFs by likewise solving Eq. (3) with respect to the set of parameters to be individualized while fixing the remaining parameter(s) to the respective nominal value(s) specified as the median value(s) of the parameter(s) derived for the 50 training subjects. We compared these ITFs in terms of their accuracy in estimating central BP waveform from diametric PVR waveforms, including the root-mean-squared waveform error (RMSE), absolute SP and PP errors (*e*_*SP*_ and *e*_*PP*_), and SP and PP error norm $(=\sqrt{{e}_{SP}^{2}+{e}_{PP}^{2}})$ across the 50 training subjects. Then, we investigated the best-performing ITF(s) in the blind testing stage.

We conducted a blind testing to investigate the performance of the best-performing GTF and ITFs as follows. First, we compared the best-performing GTF and ITFs in terms of their accuracy in estimating central BP waveform, including the root-mean-squared waveform error (RMSE), absolute SP and PP errors (*e*_*SP*_ and *e*_*PP*_), and SP and PP error norm $(=\sqrt{{e}_{SP}^{2}+{e}_{PP}^{2}})$ across the 114 testing subjects. Second, we examined the relevance of the CV risk predictors estimated from the ITFs in terms of their correlations with the reference CV risk predictors obtained directly from the carotid BP and ankle PVR waveform measurements. Third, we scrutinized the performance of the ITFs relative to the GTF with respect to different degrees of PP amplification.

### Statistical Analysis

We used the paired t-test in comparing the (1) arm TLS, arm TLG, and ankle TL GTFs; (2) f-ITF, p-ITF_1_, and p-ITF_2_; and (3) best-performing GTF and ITFs. We used a significance level of p = 0.05 with the Holm-Bonferroni correction to counteract the influence of multiple comparisons.

## Results

### Experimental Data

Table 1 shows the demographic information of the subjects analyzed in this study (median (IQR)). CV risk predictors, including BP (MP: 41–159 mmHg; DP: 28–131 mmHg), carotid-ankle PP amplification (1.01–1.62), and PTT (carotid-arm: 24–124 ms; carotid-ankle: 80–200 ms) varied widely in these subjects, indicating that the data are challenging enough to rigorously examine the validity and performance of the GTFs and ITFs under a wide range of physiological states. Comparison of CV risk predictor values associated with the training and testing as well as all data exhibits the homogeneity of characteristics (especially PTT which has a large influence on both GTF and ITF)^40^, suggesting that the findings from this study may be robust against the choice of training and testing data.

**Table 1:** Subject demographics (median (IQR)).

	Training Data (N = 50)	Testing Data (N = 114)	All Data (N = 164)
Age [Years]	58 (46–70)	56 (45–69)	57 (45–69)
Gender	M 29/F 21	M 60/F 54	M 89/F 75
Height [cm]	165 (160–168)	160 (153–167)	162 (155–167)
Weight [kg]	63 (59–72)	60 (52–68)	61 (53–69)
Systolic BP [mmHg]	116 (100–138)	115 (99–129)	115 (100–130)
Mean BP [mmHg]	90 (82–108)	93 (81–105)	93 (81–106)
Diastolic BP [mmHg]	73 (66–86)	76 (65–83)	76 (65–84)
Carotid-Ankle PP Amplification	1.12 (1.06–1.21)	1.17 (1.08–1.28)	1.15 (1.08–1.27)
Carotid-Upper Arm PTT [ms]	52 (48–56)	52 (48–56)	52 (48–56)
Carotid-Ankle PTT [ms]	136 (124–148)	136 (120–148)	136 (123–148)

### Training Results

Table 2 shows the model parameters associated with all the GTFs and ITFs considered in the training stage. In general, there was a large inter-individual variability in all the model parameters. Interestingly, *τ*_2_ exhibited the smallest variability (17.8% IQR relative to the median value), while *η*_12_ and *η*_22_ exhibited the largest variability (1216% and 291% IQR relative to the median value), compared with the rest of the parameters. Table 3 shows the performance of the GTFs and ITFs in terms of the errors associated with the estimation of central BP from diametric PVR waveforms. The arm TLS GTF overall largely outperformed its TLG counterpart and the ankle TL GTF (Table 3(a)). Both p-ITFs significantly outperformed the f-ITF (Table 3(b)): when root-mean-squared across all 50 training subjects in the training data, p-ITF_1_ and p-ITF_2_ could reduce the waveform RMSE, SP and PP errors, and SP and PP error norm significantly by 44.9% and 36.6%, 62.6% and 59.4%, 38.6% and 34.1%, and 50.0% and 46.2%, respectively, relative to the f-ITF. On the other hand, the performance of the two p-ITFs in estimating central SP and PP were statistically insignificant. These results led us to investigate the arm TLS GTF and two p-ITFs in the testing stage.

**Table 2:** Model parameters estimated from training data (median (IQR)).

	TLG GTF (Arm)	TLS GTF (Arm)	TL GTF (Ankle)	f-ITF	p-ITF_1_	p-ITF_2_
*τ*_1_	0.068	0.048	—	0.032 (0.025–0.038)	0.052 (0.040–0.060)	0.047 (0.039–0.055)
*η*_11_	0.86	20.48	—	14.54 (9.57–22.45)	14.58 (9.89–29.94)	14.45
*η*_21_	0.64	12.61	—	13.88 (9.26–20.15)	11.60 (8.77–17.94)	13.88
*E*_1_	0.81	1.43	—	0.33 (0.31–0.47)	0.33 (0.31–0.47)	0.33 (0.31–0.47)
*E*_2_	—	0.16	—	1.63 (1.44–2.02)	1.63 (1.44–2.02)	1.63 (1.44–2.02)
*η*_*VE*_	—	0.63	—	0.22 (0.19–0.26)	0.22 (0.19–0.26)	0.22 (0.19–0.26)
*τ*_2_	—	—	0.13	0.12 (0.11–0.13)	0.12	0.14 (0.12–0.15)
*η*_12_	—	—	431.3	19.3 (8.59–243.6)	189.9 (96.49–309.7)	121.6 (44.73–405.5)
*η*_22_	—	—	56.05	1.60 (0.78–5.53)	23.72 (7.29–47.98)	20.98 (3.45–64.97)

**Table 3:** Performance of the GTFs and ITFs in terms of the errors associated with the estimation of central BP from diametric PVR waveforms in training data.

Training (N=50)	RMSE [mmHg]	SP Error [mmHg]	PP Error [mmHg]	SP & PP Error Norm [mmHg]
(a) GTF				
Arm TLG GTF	4.39^*^	4.92^*^	4.35	6.57^*^
Arm TLS GTF	3.70	3.52	3.84	5.21
Ankle TL GTF	3.96	4.35	4.65	6.37
(b) ITF				
f-ITF	3.83	5.37	5.02	7.36
p-ITF_1_	2.11^†^	2.01^†^	3.08^†^	3.68^†^
p-ITF_2_	2.43^†^	2.18^†^	3.31^†^	3.96^†^

Errors are root-mean-squared across all subjects. SP: systolic pressure. PP: pulse pressure. SP & PP Error Norm: Euclidean norm of SP and PP errors. ^*^Significantly different from the arm TLS GTF (p < 0.05 with Holms-Bonferroni correction). ^†^Significantly different from the f-ITF (p < 0.05 with Holms-Bonferroni correction).

### Testing Results

**Figure 4:**
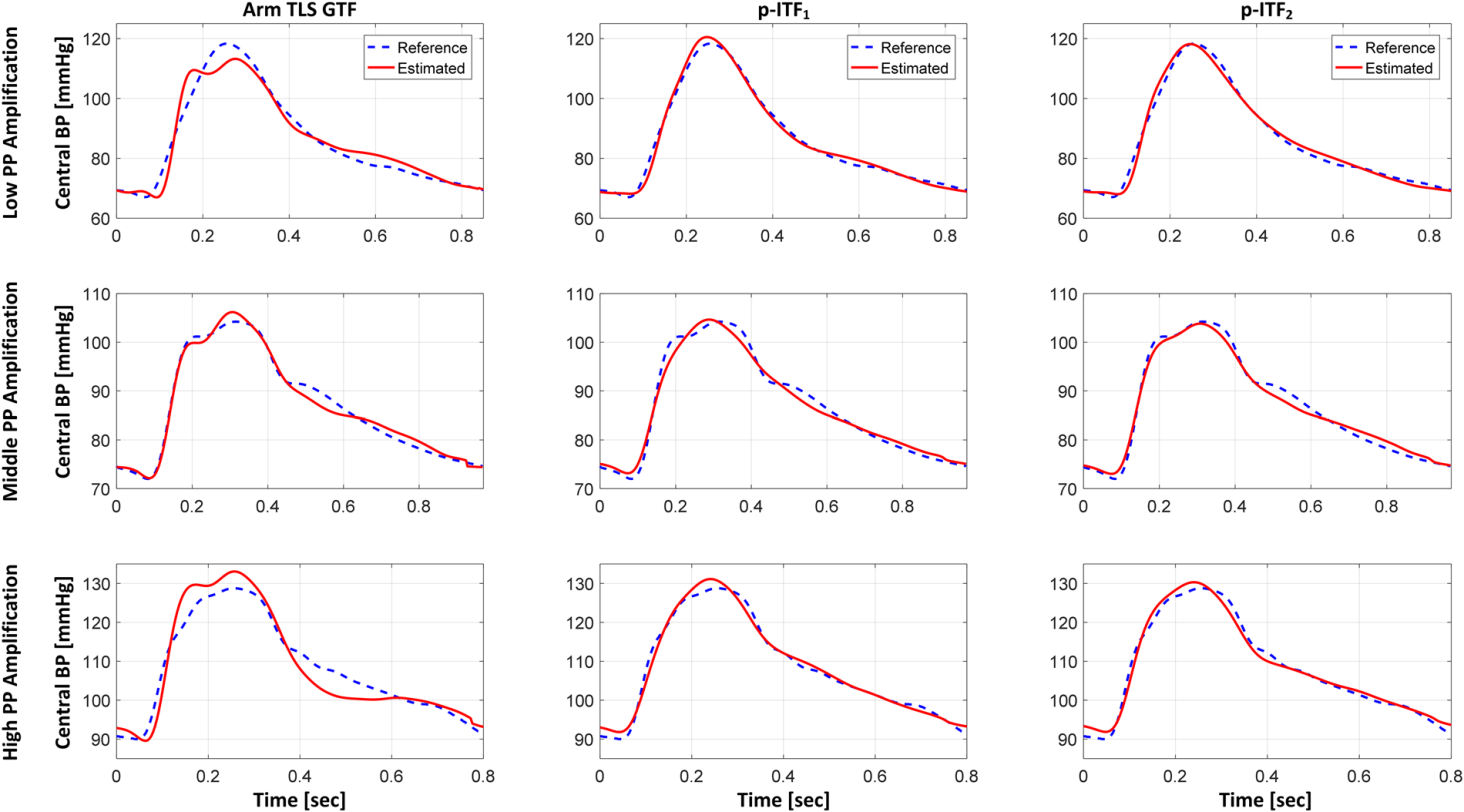
An illustrative example of central BP waveforms estimated from the arm TLS GTF, p-ITF_1_, and p-ITF_2_ under low, middle, and high PP amplification.

**Figure 5:**
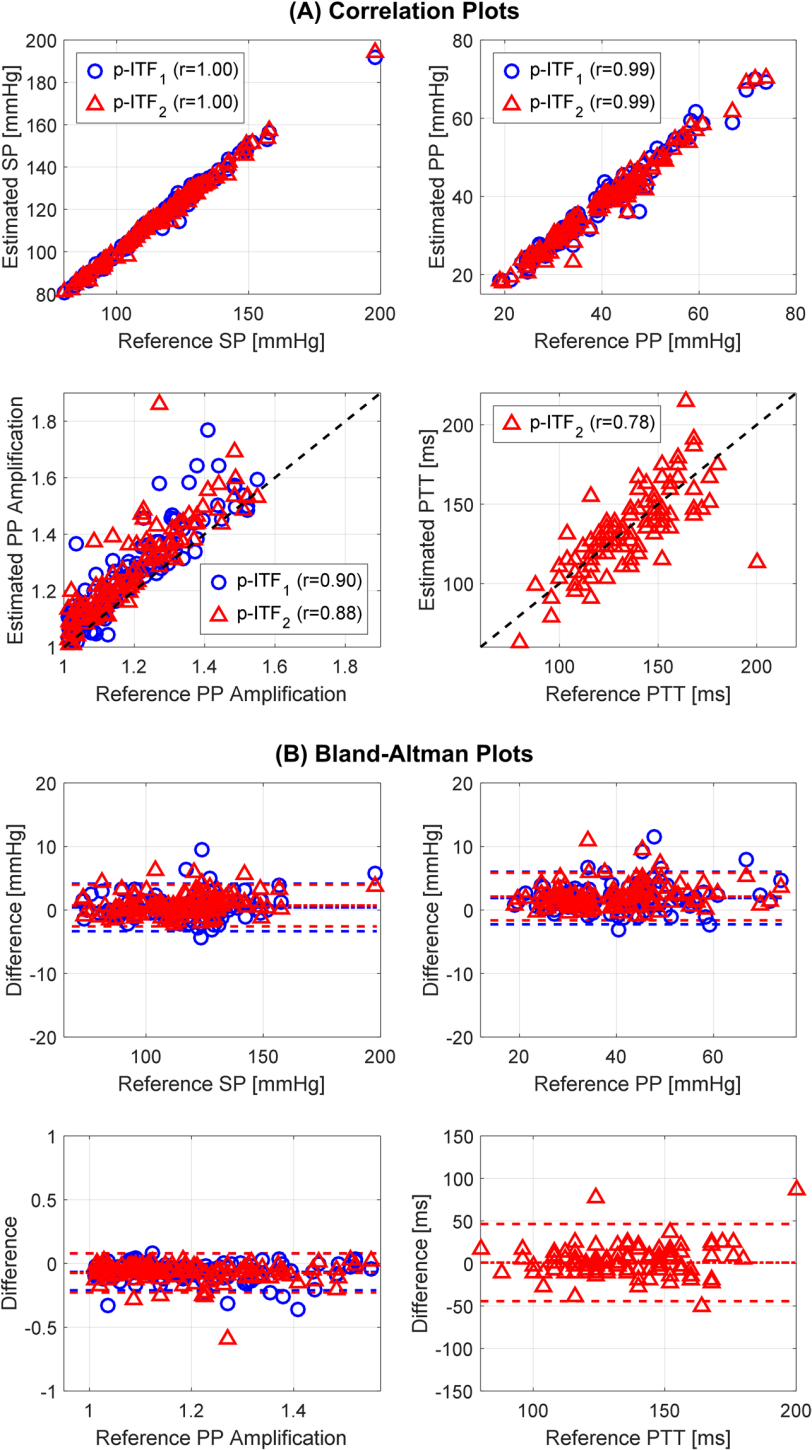
Correlation (**A**) and limits of agreement (**B**) between reference versus estimated aortic SP, PP, PP amplification, and PTT. Reference aortic SP, PP, PP amplification, and PTT are based on measured carotid BP and ankle PVR waveforms. Estimated aortic SP, PP, PP amplification, and PTT are based on central BP estimated by p-ITF and measured ankle PVR.

Table 4 shows the performance of the arm TLS GTF and two p-ITFs in terms of the errors associated with the estimation of central BP from diametric PVR waveforms, and Fig. 4 shows an illustrative example of central BP waveforms estimated from these transfer functions under low, middle, and high PP amplification. Both p-ITFs significantly outperformed the arm TLS GTF: when root-mean-squared across all 114 testing subjects, p-ITF_1_ and p-ITF_2_ could reduce the waveform RMSE, SP and PP errors, and SP and PP error norm significantly by 37.8% and 32.8%, 43.4% and 47.2%, 25.6% and 24.8%, and 32.9% and 34.1%, respectively, relative to the arm TLS GTF. Figure 5 shows the correlation and limits of agreement between reference (in terms of carotid-ankle) versus estimated aortic SP, PP, PP amplification (based on the estimated central BP and measured ankle PVR), and PTT (*τ*_2_). Both p-ITFs could estimate aortic SP (r = 1.00), PP (r = 0.99), PP amplification (p-ITF_1_: r-0.90; p-ITF_2_: r = 0.88), and PTT (p-ITF_2_: r = 0.78) that were closely correlated with their respective reference counterparts. The bias and confidence interval were also adequately small: the bias and confidence interval for aortic SP, PP, PP amplification, and PTT were 0.4% and 3.2% (p-ITF_1_) and 0.6% and 2.8% (p-ITF_2_), 4.5% and 9.8% (p-ITF_1_) and 5.0% and 8.9%(p-ITF_2_), 5.7% and 12.3% (p-ITF_1_) and 6.3% and 13.1% (p-ITF_2_), and 0.8% and 33.4% (p-ITF_2_), respectively, of the respective median values (Table 1). As a comparison, PP amplification based on the central BP estimated from the arm TLS GTF and the measured ankle PVR was significantly less correlated with the reference aortic PP amplification (r = 0.66).

**Table 4:** Performance of the arm TLS GTF and p-ITFs in terms of the errors associated with the estimation of central BP from diametric PVR waveforms in testing data.

Testing (N = 114)	RMSE [mmHg]	SP Error [mmHg]	PP Error [mmHg]	SP & PP Error Norm [mmHg]
Arm TLS GTF	3.20	3.43	3.79	5.11
p-ITF_1_	1.99^*^	1.94^*^	2.82^*^	3.43^*^
p-ITF_2_	2.15^*^	1.81^*^	2.85^*^	3.37^*^

Errors are root-mean-squared across all subjects. SP: systolic pressure. PP: pulse pressure. SP & PP Error Norm: Euclidean norm of SP and PP errors. ^*^Significantly different from the arm TLS GTF (p < 0.05 with Holms-Bonferroni correction).

Table 5 shows the performance of the arm TLS GTF and two p-ITFs in subjects associated with low, middle, and high PP amplification. The superiority of the p-ITFs to arm TLS GTF was all in all consistent across all the PP amplification regimes, but especially in low and high PP amplification regimes. When root-mean-squared across all 38 testing subjects with low PP amplification, p-ITF_1_ and p-ITF_2_ could reduce the waveform RMSE, SP and PP errors, and SP and PP error norm significantly by 38.6% and 35.9%, 39.1% and 46.3%, 22.5% and 26.6%, and 29.5% and 34.8%, respectively, relative to the arm TLS GTF. When root-mean-squared across all 38 testing subjects with high PP amplification, p-ITF_1_ and p-ITF_2_ could reduce the waveform RMSE, SP and PP errors, and SP and PP error norm significantly by 36.8% and 28.9%, 55.1% and 67.6%, 41.6% and 50.5%, and 46.8% and 56.8%, respectively, relative to the arm TLS GTF.

**Table 5:** Performance of the arm TLS GTF and p-ITFs in subjects associated with low, middle, and high PP amplification.

Testing (N = 114)	Low (1.02–1.30)				Middle (1.31–1.38)				High (1.38–1.58)			
	RMSE	SP Error	PP Error	Norm	RMSE	SP Error	PP Error	Norm	RMSE	SP Error	PP Error	Norm
Arm TLS GTF	3.77	4.67	5.15	6.96	2.94	2.22	2.15	3.09	2.80	2.91	3.46	4.52
p-ITF_1_	2.32^*^	2.85^*^	3.99	4.90^*^	1.84^*^	1.24^*^	1.96	2.32	1.77^*^	1.30^*^	2.02^*^	2.41^*^
p-ITF_2_	2.42^*^	2.51^*^	3.78	4.54^*^	2.01^*^	1.64^*^	2.65	3.12	1.99^*^	0.94^*^	1.71^*^	1.95^*^

Errors are root-mean-squared across all subjects. SP: systolic pressure. PP: pulse pressure. Norm: Euclidean norm of SP and PP errors. ^*^Significantly difference from the arm TLS GTF (p < 0.05 with Holms-Bonferroni correction).

## Discussion

We presented and demonstrated the validity of a novel multiple measurement information fusion approach to non-invasive estimation of CV risk predictors from PVR waveforms measured at diametric locations in the body. This approach has a few advantages compared with the state-of-the-art techniques for estimation of CV risk predictors. First, it may be more accurate than the traditional GTF approach by virtue of subject specificity. Second, it may offer more comprehensive assessment of CV risk in a subject by virtue of its ability to estimate a number of CV risk predictors. Third, it may enable convenient monitoring of CV risk predictors by obviating the measurement of central pulse waveform (which usually requires costly procedures and trained operators).

The proposed p-ITFs (which significantly outperformed the f-ITF in the training stage) boasted significantly superior performance to the arm TLS GTF (which was the best performing GTF obtained in the training stage) in estimating central BP waveform in blind testing (Table 4). In terms of enabling non-invasive estimation of central BP waveform, this study can be viewed as a leap from a series of our prior work, where we have shown that an f-ITF based on invasive diametric arterial pulse waveforms could estimate central BP waveform more accurately than an arm TL GTF^10,27^. In this study, we found that the f-ITF did not perform as well as the arm TLS GTF when realized with diametric PVR waveforms (Table 3), which may be attributed to two reasons: (1) PVR waveforms are less informative than arterial BP waveforms with the high-frequency contents in the latter lost in the former due to arterial and tissue viscoelasticity, and (2) non-invasive f-ITF involves more complex arterial line models with increased number of parameters than its invasive counterpart, making it more susceptible to overfitting when used with PVR waveforms offering relatively sparse information contents. We also showed that partial individualization of the arterial line models (i.e., the p-ITFs) could largely enhance the performance of the ITF, perhaps via the regularization of the parameter estimation process for the arterial line models to prevent overfitting. It is important to emphasize that the p-ITFs considered in this study are built upon solid rationale and insight. First, the p-ITF_1_ was motivated by the observation from Table 2 that *τ*_2_ showed the smallest inter-individual variability among all the arterial line model parameters and may thus be fixed at a nominal value. Second, the p-ITF_2_ was motivated by the widely accepted knowledge that the arterial line model exhibits much larger sensitivity to its PTT parameter *τ*_*j*_ than the polynomial parameters *η*_*ij*_, *i*, *j* = 1, 2^10,27^. According to Table 2, the ankle line polynomial parameters (*η*_12_ and *η*_22_) showed larger inter-individual variability than the arm line polynomial parameters (*η*_11_ and *η*_21_). Further, our prior work indicates that *η*_11_ and *η*_21_ are subject to larger estimation uncertainty than *η*_12_ and *η*_22_^10,27^. Hence, it made a lot of sense to regularize the parameter estimation by fixing *η*_11_ and *η*_21_ to appropriate nominal values in considering viable options for p-ITF. Therefore, it was not surprising that p-ITF_1_ and p-ITF_2_ examined in this study performed very well. However, it must be noted that the efficacy of p-ITF_1_ may not generalize to subjects whose PTT drastically deviate from the population-average nominal value (e.g., in subjects receiving vasoactive agents), whereas p-ITF_2_ may still generalize well to a wider range of subjects.

The proposed p-ITFs (p-ITF_2_ in particular) could estimate a number of CV risk predictors solely based on non-invasive diametric pulse measurements (i.e., without requiring any direct measurement of central pulse waveform): aortic SP, PP, PP amplification, and PTT. These CV risk predictors were estimated from the individually estimated arterial line models (i.e., *τ*_2_ as aortic PTT^36)^ and central BP waveform (i.e., peak and pulse amplitude of the estimated central BP waveform as aortic SP and PP) in conjunction with the measured PVR waveform (i.e., ratio between the amplitudes of estimated central BP and ankle PVR waveforms as aortic PP amplification). Encouragingly, the CV risk predictors thus estimated were closely correlated with the reference CV risk predictors derived from carotid BP and ankle PVR waveform measurements with tight limits of agreement (Fig. 5). In our prior work, we showed that an ITF based on invasive arterial BP waveforms could track the time-varying PTT in a subject^27,30^. The results obtained from this study suggest that aortic PTT and other CV risk predictors may be monitored conveniently through time. Currently, non-invasive measurement of aortic BP, PP amplification, and PTT resorts to carotid-femoral tonometry procedure, which requires costly equipment and trained operators to measure both central and distal arterial pulse waveforms. Technologies to incorporate the functionality to estimate central BP from arm PVR waveform into today’s arm cuff devices exist^21–25^. However, these technologies have limitations in estimating aortic PP amplification and PTT due to the absence of distal aortic pulse measurement. In this regard, obviating the measurement of central pulse waveform in estimating a range of CV risk predictors may be viewed as a significant innovation of this study. In fact, the proposed approach may be readily integrated with already available medical devices (e.g., dual arm-ankle cuffs or a pair of finger-toe pulse oximeters) to enable convenient out-of-clinic monitoring of CV risk predictors. That being said, it must also be emphasized that arm cuff device equipped with a GTF may still be a convenient option for brachial and central BP measurement, if CV risk predictors requiring the measurement of distal aortic pulse is not of primary interest.

Scrutinizing the performance of the arm TLS GTF and p-ITFs obtained from the blind testing with respect to the degree of PP amplification, the p-ITFs were superior to the arm TLS GTF independently of PP amplification. In particular, the p-ITFs were significantly superior to the arm TLS GTF in all error metrics in subjects with low and high PP amplification regimes (except the PP error in low PP amplification regime). The difference between the p-ITFs and the arm TLS GTF was less significant in the middle PP amplification regime, which may be attributed to the fact that the arm TLS GTF was trained to perform adequately in all the PP amplification regimes, and thus, it is expected to perform relatively better in subjects with middle than low and high PP amplification.

As an additional remark, it must be emphasized that the arm TLS GTF is a secondary contribution of this study regardless of the focus of this study on the ITF. In fact, we showed in our prior work that, on an individualized basis, arm TLS model outperformed its TLG counterpart and individualized ankle TL model in estimating central BP waveform from the respective distal PVR waveforms^37^. The results obtained in this study (Table 3(a)) show that our previous findings persist even on a generalized basis. Noting that (at least a subset of) prior work on the GTF resorted to the assumption that arm PVR waveform may be used as a surrogate of brachial BP waveform^19^, the arm TLS GTF may offer opportunities to improve the efficacy of GTF in estimating central BP waveform.

This study has a few limitations. First, we examined only two options for the p-ITF. On one hand, the two p-ITFs were built upon solid rationale and insight, and for that reason, were shown to perform very well. On the other hand, there are many alternatives to construct p-ITF that could have been explored. In this regard, more extensive investigation of the p-ITF approach may be a rewarding exercise. Second, we examined only the use of a pair of PVR waveforms from arm and ankle for the realization of the proposed approach. Other distal sites such as ear, finger, and toe may afford viable options for practical implementation of the approach. In addition, the efficacy of the approach may benefit from increasing the number of measurements, e.g., by leveraging richer information contents to robustify the ITFs. Future work must explore such opportunities. Third, the ethnic group we examined was rather homogeneous (Asians). Future work must examine the performance of the proposed approach in diverse ethnic groups in order to truly establish its efficacy.

In summary, we demonstrated the efficacy of the proposed multiple measurement information fusion approach in estimating a range of CV risk predictors (including central SP and PP as well as aortic PP amplification and PTT). The CV risk predictors estimable from the proposed approach have already been shown to have close relationship to CV health and disease (in case of central SP and PP as well as aortic PP amplification) and other established CV risk predictors (in case of aortic PTT in terms of carotid-ankle PTT, which is correlated with carotid-femoral PTT). Hence, the close correlation between the CV risk predictors estimated from the proposed approach and the reference CV risk predictors (Fig. 5) suggests that the CV risk predictors derived from the proposed approach may be valuable in predicting CV health, risk, and disease more conveniently than the state-of-the-art carotid-femoral tonometry procedure.

Yet, the strict predictive power of the proposed approach for CV risk must be validated using longitudinal data of the patients with history of CV events. That said, the proposed approach may complement the prior analyses and models based on the existing large-scale studies (including, e.g., the Framingham Heart Study) as well as benefit the design of future large-scale studies on CV health, risk, and disease. First, it may compliment the prior analyses and models with novel subject-specific CV risk predictors. Specifically, considering that (1) a subset of prior studies found limited efficacy of the GTF-estimated central SP and PP as well as PP amplification based on radial (i.e., non-aortic) tonometry in predicting CV risk^41^, and that (2) the ITF-estimated subject-specific central SP and PP as well as aortic PP amplification correlated closely to the reference CV risk predictors, the CV risk predictors derived from the ITFs may be exploited to improve the predictive power of the prior analyses and models. In addition, despite its close correlation to the gold standard carotid-femoral PTT, the efficacy of carotid-ankle PTT in CV risk prediction has yet to be rigorously investigated in future work. In this way, the proposed approach has potential to enrich and complement today’s CV risk prediction paradigm. Second, it may benefit the design of future studies on CV health and disease as an analytic tool capable of simplifying the instrumentation protocols. For example, the proposed approach may be leveraged to obviate the need for carotid artery tonometry procedure in the measurement of central SP, PP, and carotid-femoral PTT performed in prior large-scale studies^42^. In addition, it may even completely eliminate the need for arterial tonometry procedure (especially at the femoral artery, which can entail discomfort in many subjects due to the access to private body site) in the measurement of central SP and PP as well as aortic PP amplification and PTT^43,44^ with its potential to derive equivalent CV risk predictors simply based on the diametric PVR waveform measurements. Future investigations to explore the potential of the proposed approach is thus worth pursuing.
